# AFLA-PISTACHIO: Development of a Mechanistic Model to Predict the Aflatoxin Contamination of Pistachio Nuts

**DOI:** 10.3390/toxins12070445

**Published:** 2020-07-10

**Authors:** Michail D. Kaminiaris, Marco Camardo Leggieri, Dimitrios I. Tsitsigiannis, Paola Battilani

**Affiliations:** 1 Laboratory of Plant Pathology, Department of Crop Science, School of Plant Sciences, Agricultural University of Athens, Iera Odos 75, 11855 Athens, Greece; mikekaminiaris@gmail.com (M.D.K.); dimtsi@aua.gr (D.I.T.); 2 Department of Sustainable Crop Production (DI.PRO.VE.S.), Universita Cattolica del Sacro Cuore, Via Emilia Parmense 84, 29122 Piacenza, Italy; marco.camardoleggieri@unicatt.it

**Keywords:** aflatoxin B_1_, probability, weather, *Aspergillus flavus*, preharvest, model transfer

## Abstract

In recent years, very many incidences of contamination with aflatoxin B_1_ (AFB_1_) in pistachio nuts have been reported as a major global problem for the crop. In Europe, legislation is in force and 12 μg/kg of AFB_1_ is the maximum limit set for pistachios to be subjected to physical treatment before human consumption. The goal of the current study was to develop a mechanistic, weather-driven model to predict *Aspergillus flavus* growth and the AFB_1_ contamination of pistachios on a daily basis from nut setting until harvest. The planned steps were to: (i) build a phenology model to predict the pistachio growth stages, (ii) develop a prototype model named AFLA-pistachio (model transfer from AFLA-maize), (iii) collect the meteorological and AFB_1_ contamination data from pistachio orchards, (iv) run the model and elaborate a probability function to estimate the likelihood of overcoming the legal limit, and (v) manage a preliminary validation. The internal validation of AFLA-pistachio indicated that 75% of the predictions were correct. In the external validation with an independent three-year dataset, 95.6% of the samples were correctly predicted. According to the results, AFLA-pistachio seems to be a reliable tool to follow the dynamic of AFB_1_ contamination risk throughout the pistachio growing season.

## 1. Introduction

Pistachio (*Pistacia vera* L.) is a dioecious wind-pollinated tree cultivated for its highly nutrient nuts in many regions of the world under different climate conditions, but especially in subtropical and temperate zones [1]. Warm summer and mild winter conditions are preferable to achieve optimum yields and a high-quality product. It is well known that in order to attain high yields, chilling accumulation is essential during winter months, specifically from early October until late March [2,3], and hot conditions from April until harvest [4]. Several models exist to quantify the chilling accumulation, such as the Dynamic Model (in Chill Portions (CP)), which was originally developed for warm winters. It assumes that winter chill results from a process that takes place in two steps, in which an intermediate product is firstly formed in a process promoted by cold temperatures. Warm temperatures can destroy this intermediate product. As soon as a specific quantity of this intermediate has accumulated, it is irreversibly transformed into a Chill Portion, which can no longer be destroyed [5]. Furthermore, there is the Chilling Hours (CH) Model, which is driven by the Crossa-Raynaud Model and the Utah Model (Utah Chill Units (UCU)) [5]. The Chilling Hours Model is the oldest method available to quantify winter chill and is still widely used. It considers all the hours with temperatures between 0 and 7.2 °C as equally effective for winter chill accumulation [5]. Likewise, models for heating accumulation have been used for crop growth stage description, such as Growth Degree Days (GDD) [6,7]. The basic equation used is GDD = [(T_Max_ + T_Min_)/2] − T_Base_, where T_Max_ and T_Min_ are the daily maximum and minimum air temperature, respectively, and T_Base_ is the base temperature, also cited as threshold [7].

In Europe, pistachio nut trees are mainly cultivated around the Mediterranean basin. Greece is the 6th top pistachio producing country worldwide (FAOSTAT 2012), with an annual total production of around 10,000 tons. The Greek variety *Pistachia vera* cv *Aegina* has been traditionally cultivated from the beginning of the 20th century, and the nuts produced are registered as Product of Designation of Origin (PDO). Due to the high added value of PDOs, pistachio nut cultivation plays a significant role in boosting the local economy of the growing areas.

Pistachio nuts are very often contaminated by aflatoxins (AFs), naturally occurring toxic compounds produced by fungi belonging to *Aspergillus* section *Flavi*, with a major role attributed to *A. flavus* in the USA [8,9,10], Iran [11] and also Greece [12]. Aflatoxins, aflatoxin B1 (AFB_1_) in particular, are the most hazardous mycotoxins, with confirmed carcinogenic, genotoxic, and immunosuppressive effects for humans [13]. They can be detected in several agricultural products, mainly maize and nuts at preharvest level, but the contamination can increase significantly at postharvest with the presence of contaminated kernels and the use of improper nut storage management conditions [14,15,16]. Therefore, prevention is mainly based on rational crop management in field, but with care in postharvest to ensure prompt drying methods and storage in non-conducive conditions for *Aspergillus* nut spoilage and aflatoxin contamination [17]. 

The periods from flowering until setting and also maturity, in particular hull splitting, are considered to be the susceptible growth stages for *A. flavus* infection. In the period from flowering until setting, the fungus can penetrate into the setting nut and colonize the internal part of the nut, the hard shell not being yet present. Furthermore, nut shell splitting during pistachio maturity is considered to be a very susceptible stage; delayed harvest can lead to high levels of aflatoxin accumulation due to favorable conditions for *A. flavus* infection in nuts through the split shell. Additional studies also correlate high levels of aflatoxin with insect-damaged and wounded nuts [10,18].

Harvesting time is considered to be a very critical parameter for high-quality products free of aflatoxins [19]. The moisture content and water activity (a_w_) of nuts differ depending on the time of harvesting; the moisture content is usually between 32% and 38% [20], whereas the a_w_ remains very high until harvesting, at around 0.99 [21]. After harvest, mechanical dehulling, intended to separate the soft hull from the shell, takes place in water dehullers [12]; this is crucial in order to avoid the oxidation of the hull and consequently the blackening and decay of the shelled nuts. After dehulling, immediate drying follows to achieve a nut moisture content of around 12–13%. Concerning a_w_, 0.82 or lower values are required for short-term storage and 0.7 or lower for long-term storage [20,22]. Drying can be achieved either mechanically in hot-air dryers (65–70 °C for about 8–10 h) or in the sun by spreading pistachio nuts in a thin layer of 2–3 cm thickness for approximately 2–3 days. Immediate drying using hot-air dryers is of ultimate importance in order to avoid aflatoxin production due to favorable conditions for *A. flavus*.

Legal limits have been set for the maximum aflatoxin content in several agricultural products, nuts included, by the European Union (EU Reg EC 1881/2006, as amended by 165/2010) in order to prevent consumer exposure. Therefore, uncompliant production is not suitable for the market, with a great impact on farmers’ incomes. The significance of the economic impact of aflatoxin occurrence in agricultural products has been highlighted by a social network analysis of commercial exports and global trade patterns [23].

Good management practices at the postharvest level are clearly defined and they can guarantee no aflatoxin increase if properly applied. Therefore, during recent years, research has been focused on the prevention of aflatoxin contamination in pistachio fields using biological or chemical plant protection products [24,25,26,27]; nevertheless, no predictive models are available to support farmers’ decisions in pistachio crop management. Predictive models have widely been used in Integrated Pest Management (IPM) systems of plant diseases in the frame of sustainable crop production, and several examples are available for mycotoxin management, such as the ochratoxin A (OTA) contamination of grapes [28], the fumonisin contamination of maize [29], the aflatoxin contamination of peanuts [30], and the deoxynivalenol contamination of winter wheat [31,32,33]. In the context of the present work, the most interesting model from the literature is AFLA-maize, a mechanistic model to predict *A. flavus* growth and AFB_1_ contamination risk in maize [34,35]. It is based on two sub-models, one accounting for the host crop phenology and the other for the *A. flavus* infection cycle. The rationale behind the present study was to transfer the available knowledge, intended as the predictive maize model of *A. flavus* and aflatoxins, to a different pathosystem, *A. flavus*-pistachio. In particular, the objectives of this study were to (i) develop a predictive model for pistachio phenology in order to be combined with the sub-model of *A. flavus* infection cycle; (ii) define the susceptible pistachio growth stage for *A. flavus* infection; (iii) develop and validate the resulting predictive model with the aflatoxin data collected from Greek pistachio orchards.

## 2. Results

### 2.1. Collection of Meteorological Data

The collected meteorological data were: the daily mean temperature (T, °C), maximum daily temperature (T_max_, °C), minimum daily temperature (T_min_, °C), daily relative humidity (RH, %), and daily precipitation (R, mm) from the 1st of January until the 31st of December for the years 2014–2019. The collected meteorological data were used to compute the pistachio growth stages (2014–2016) and to develop and validate the predictive model, AFLA-pistachio (2017–2019).

### 2.2. Computation of Pistachio Growth Stages

The island of Aegina is a warm and dry area where the daily minimum temperature during winter is above 7 °C, so there is no chilling accumulation (www.meteo.gr). As a result, the CH calculation for pistachio trees using the Crossa-Raynaud model was proved to be not applicable for the region of Aegina island due to high winter temperatures. 

The GDD model was applied for the determination of the growth stages of the crop. The GDD based on the field observations for 2014, 2015, and 2016 were calculated for all the crucial growth stages of the crop (Table 1). 

For all the three years, the computation of GDD indicated that flowering occurs after at least 579 GDD and varies up to 836 GDD, with a mean daily temperature (T) in this period of 17.9 °C (mean of three years); 2015 had the lowest temperatures, while 2014 and 2016 were warmer years. Consequently, in 2015 all the growth stages occurred later than in 2014 and 2016. Harvesting takes place around 3000 GDD for the current location investigated, specifically 3013 GDD, 2845 GDD, and 3167 GDD for 2014, 2015, and 2016, respectively. The existence of small period windows leads to the result that the differentiation between the years studied occurs within a small frame.

### 2.3. Field Sampling and Aflatoxin Occurrence Data

Twenty-nine pistachio samples were analyzed for AFB_1_ contamination in 2014, 11 in 2015, 3 in 2016, 54 in 2017, 20 in 2018, and 13 samples in 2019. For example, in 2014 the AFB_1_ contamination fluctuated from 0.2 to 180 μg/kg (mean 22 μg/kg), and in 2015 from 0.1 to 73 μg/kg (mean 18 μg/kg). Contamination data are presented analytically in Table 2; the contamination data from each orchard sample were compared to the prediction of the model for that specific weather station and year. The percentage of field samples exceeding the legal limit of AFB_1_ contamination set at 12 μg/kg was 40% for 2014, 31% for 2015, and 2% for 2017, and no samples exceeded the legal limit in 2016, 2018, and 2019.

### 2.4. Predictive Model

**Figure 1:**
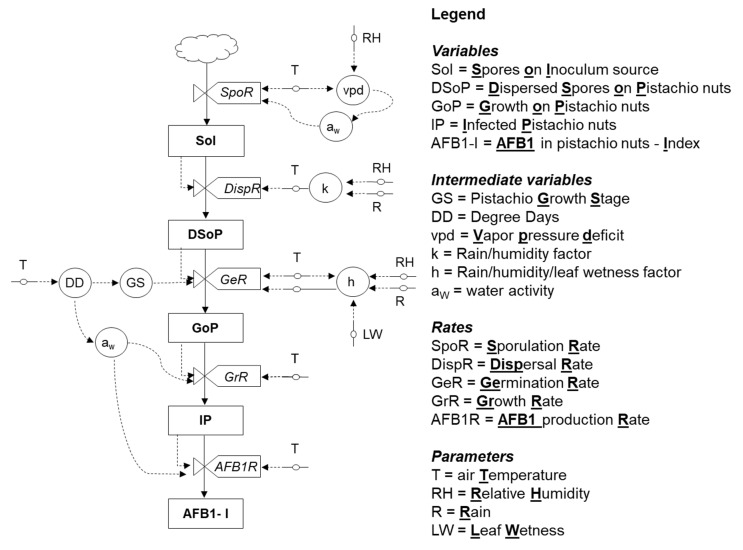
Relational diagram of the predictive model for *Aspergillus flavus* growth and aflatoxin production in pistachio nuts.

The relational diagram (Figure 1) that describes the infection cycle of *A. flavus* on pistachio starts with the inoculum source, which is not quantified in the model. The produced spores (SoI) are dispersed (DispR) and they arrive on pistachio bunches (DSoP). The spores’ flux is described by a dispersal rate (DispR). When the environmental conditions are suitable, the spores germinate, and the fungus can grow (GoP) and infect (IP) early-developed nuts according to the germination and growth rate (respectively, GeR and GrR). If pistachio is in a susceptible growth stage (GS), from flowering to splitting the fungal growth continues in pistachio nuts, as well as AFB_1_ production (AFB_1_-I), regulated by the AF production rate (AFB_1_R). 

The AFB_1_ cumulative index AFB_1_-I, intended as the AFLA-pistachio output, was computed using the meteorological data as input from flowering to crop harvest (Table 2). For the considered years (2014–2016), the AFB_1_-I varied between 890 (minimum value computed in 2014) and 1257 (maximum value computed in 2015); the mean AFB_1_-I values were 1014 and 1043, respectively, in 2014 and 2015. 

### 2.5. Probability of Aflatoxin Contamination

According to the logistic regression, parametrized with dataset 1 using the AFB_1_-I calculated by the predictive model as the independent variable (Table 3), the probability *p* = 0.5 corresponded to AFB_1_-I = 1153. Comparing the predicted not contaminated samples with the observed data for the internal validation, intended as each field sample collected in the 3-year period 2014–2016 (43 samples in total, Table 4), 55.8% of the prediction was correct (predicted 0 and observed 0), while 16.3% was a false negative prediction (predicted 0 and observed 1). Regarding the prediction of contaminated samples, 16.3% were correctly predicted (predicted 1 and observed 1) and 11.6% were overestimated (predicted 1 and observed 0). The global accuracy of the model was 72.1% (55.8% + 16.3%). The accuracy refers to the percentage of individual samples per year as the model denoted the potential risk for contamination. The robustness of the model is indicated through the percentage of correctly predicted samples; the higher the number of correctly predicted samples, the higher the robustness of the model.

### 2.6. External Model Validation

In dataset 2 (2017–2019, Table 4), 98.9% of the samples were classified as not contaminated (AFB_1_ content in pistachio nut lower than 12 µg/Kg), and the remaining 1.1% were classified as contaminated. According with the probabilistic equation developed, 96.7% of the samples were predicted as not contaminated and 3.3% as contaminated. The contingency table shows that 95.6% of the samples were classified as not contaminated (predicted 0 and observed 0) and none as contaminated (predicted 1 and observed 1). Almost 3% of pistachio nut samples were overestimated (predicted 1 and observed 0), and only 1.1% of samples were underestimated (predicted 0 and observed 1). The global accuracy of predictions with dataset 2 not used for the development of the probabilistic function was 95.6%.

## 3. Discussion

Predictive models are nowadays very useful tools supporting modern IPM for managing plant pathogens and the mycotoxins they produce [36]. Pistachio nuts are one of the commodities contaminated by aflatoxins [12], and the meteorological and contamination data suggest that the Mediterranean basin is an area highly prone to aflatoxin contamination. Global warming is also significantly altering the distribution of temperature variability and extremes and precipitation patterns, with a significantly higher frequency of exceptionally unfavorable years for several crops. Therefore, it is very important to organize a predictive system for AF risk in pistachios especially under climate change scenarios, as recently stressed by Battilani and Camardo Leggieri [34] for aflatoxins in maize. Therefore, research is focusing on preharvest, this being the growing period of crops crucial to determine “if” mycotoxin contamination will take place and “how high” it will be at harvest [37]. As a result, supporting producers via predictive models is the ultimate goal of the new generation decision support agricultural systems.

Despite the fact that pistachio cultivation is considered to be a minor crop in Greece as well as in several other regions of the world, the importance of the crop is underlined by the high added product value for producers. However, pistachio farmers often face an aflatoxin contamination problem. As a result, this problem not only leads to uncertainty in the consumer perception of consuming pistachios, but also creates additional production costs and income loss for producers, distributors, and other stakeholders.

In the frame of the current study, the phenology of pistachio nut cultivation was investigated. The crop phenology of pistachio cultivation appears to have been insufficiently studied. The lack of recorded data imposes the need for research regarding the tree’s phenology.

The growth degree day approach, as described in the literature [38,39], was selected to describe the tree’s phenology in the current study using on site observations combined with meteorological data. A model for crop phenology was developed; it worked reasonably well for several consecutive years for Aegina island, with a day interval of 1.4–4.0 days in the first 2 years and one a bit wider in the third year (4.0–6.8 days). Therefore, the pistachio phenology can be properly predicted as support for the predictive model.

The pistachio variety Aeginis, widely cultivated in Greece, appears to be better adapted to higher winter temperatures and lower chilling accumulation when compared to other pistachio varieties such as Kalle-Ghuchi and the Tunisian cultivar Mateur [39,40,41]. This is also in accordance with data regarding the chilling requirements of pistachio trees in California’s Central Valley, where trees are not adapted to such high winter temperatures as in Greece [42].

In the current work, the developed model for the phenology of the crop was combined with an already existing model for the prediction of the risk of *A. flavus* infection in maize and aflatoxin contamination above the legal limit [33]. Specifically, the model accuracy in the model development and internal validation was 72.1%, with 16.3% underestimates and 11.6% overestimates. This is comparable with the predictive model performances in other pathosystems, such as *Fusarium* spp in wheat [31] and *A. flavus* in maize [33,43].

The model performance exceeds the accuracy of 95.6% correctly classified samples in the external validation, with 3.3% of pistachio nut samples overestimated and only 1.1% of samples underestimated. This result is very good, considering that AFLA-pistachio is a mechanistic model using as input only meteorological data. However, it is important to highlight that a higher number of samples contaminated above the legal limit should be used in order to confirm the robustness of the developed model.

The AFB_1_-I values given by the model varied from year to year, which leads to the conclusion that the index is sensitive to changes in weather conditions. The model demonstrated the highest AFB_1_-I values, specifically 1014 and 1043, in years 2014 and 2015, respectively, whereas in 2016 the AFB_1_-I was 879. The warmest year of the three of dataset 1 was 2016. On the other hand, the data for the period 2017–2019, which have been used as dataset 2 for external validation (comparison between the model predictions and real observations of AF contamination), led to lower AFB_1_-I values, varying from 349 to 900. The coldest year of all the 6 years studied was 2019, and it also had the lowest model AFB_1_-I value (AFB_1_-I = 349). Based on these numbers, it is clear that the perception of warmer year alone is not sufficient to support a high aflatoxin contamination risk; the predictive model confirms its role in supporting the risk prediction.

The threshold for the classification of the samples is set at 12 µg/kg of AFB_1_, which is the legal limit for pistachio nuts destined for human consumption. Based on a three-year external validation dataset, the AFLA-pistachio model performance (95.6%) is considered to be high compared to other existing developed models for mycotoxin contamination prediction, like AFLA-maize with 73% accuracy [33] or 84% accuracy for the prediction of deoxynivalenol in wheat [44], taking into consideration that the validation was conducted based on a 3-year contamination dataset. It has also been well demonstrated that several factors, such as the crop variety and its characteristics, can affect aflatoxin contamination [45]. In the current research, such factors were not taken into consideration and were not included in the development of the model because the variety was common to all the orchards assessed. Although the existing inoculum of *A. flavus* in each pistachio orchard may vary from field to field, this factor was neither taken into consideration nor included in the model development. This is because the presence of *A. flavus* inoculum in the field is a guarantee that contamination is possible and also because of the difficulty and uncertainty in its assessment in each orchard. Additionally, it is important to underline that agricultural practices can significantly impact the fungal growth and mycotoxin accumulation in the complex interactions in the host plant-fungi-environment, as stated for maize [46] and wheat [47]. High interaction between all the agricultural practices applied can make it very difficult to understand the effects of each operation and even harder to model it with mathematical functions in order to be included in a mechanistic model [37]. In fact, also in the most studied pathosystem related to mycotoxin, *Fusarium* head blight in small grains, “agronomic programmes” or “arrays of growing techniques” are mentioned, more than single actions, to explain the risk of contamination [48,49]. Empiric approaches were instead used to account for the cropping system in maize [50].

Depending on the agronomical characteristics of each variety cultivated, the early split of pistachio nuts can be controlled up to a certain level by the application of good agricultural practices such as appropriate irrigation. After the naturally occurring split shell, during the maturity stage of the nuts the crop enters a susceptible period where the nuts are more prone to be aflatoxin-contaminated. As the model output provides a daily prediction, it is clearly shown that the date of harvest is a crucial factor affecting the aflatoxin contamination of pistachio nuts. The model predicts a high-risk condition, and therefore it indicates the appropriate time of harvest, which is a very useful suggestion for farmers. This can reduce the aflatoxin contamination of pistachio nuts at preharvest and also indicate the lots with the highest risk of contamination due to the field prediction which require more careful postharvest management.

As such a type of support has been becoming more available to farmers during recent years, the application of agronomical practices and plant protection products can be guided by predictive models. This guidance can be achieved with Decision Support Systems (DSS) that exploit the output of predictive models and provide farmers with valuable information [51]. The model developed in this study could be the core of a DSS, aimed at improving pistachio nut cultivation and reducing the inflow of chemicals in the food chain production. This is crucial, nowadays, in the widest context of IPM, which is fundamental for ensuring agricultural productivity while maintaining economic and environmental sustainability [52,53].

As far as we know, this is the first attempt to transfer such a mechanistic model from one crop to another sharing problems caused by a common fungus. The results are very encouraging, with a model accuracy comparable to the original model developed for *A. flavus* in maize. The external validation was excellent, but both the limited number of official samples obtained for the validation in the 3-year period 2017–2019 (87 in total) and their distribution respect to the fixed threshold (all except 1 below the contamination threshold) make this result not conclusive. Nevertheless, the model transfer apparently works; this preliminary validation must be supported by further data to state the robustness of the model before its delivery as a support tool for farmers. Further, model transfer may lead, in the future, to the association of the *A. flavus* sub-model with other crops of interest for aflatoxin contamination. This should be considered a valuable asset in the prevention of the mycotoxin contamination of crops and food products, with the practical application of preharvest models also in areas with a high potential risk of mycotoxin contamination but poor resources for research and modelling.

## 4. Materials and Methods

### 4.1. Collection of Meteorological Data

The daily meteorological data of air temperature (T, °C) and rain (R, mm) were collected from the website www.meteo.gr, supported by the National Observatory of Athens for Aegina island, a region with considerable PDO pistachio production in Greece. The daily relative humidity (RH, %) data were kindly supplied by the Greek National Meteorological Service. The island is located close to Athens in the prefecture of Piraeus, region of Attica, in the Saronic Gulf. The geographical coordinates of Aegina city, the capital of the island, are 37°44′51.42′’ and 23°25′44.97′’. The reference period was 2014–2019.

### 4.2. Description and Computation of Growth Stages

The chilling hours were calculated based on the Crossa-Raynaud Model [1], the most commonly used model for the computation of pistachio growth stages; the data from 1st October to 31st March in the seasons 2013–2014, 2014–2015, and 2015–2016 were used.

A very crucial point when using this model for the description/prediction of crop phenology is the definition of the temperature threshold; for pistachio trees, the threshold is defined at 7 °C and a temperature below 7 °C is required during winter for the break of dormancy for most varieties [40,54].

The chilling period is then followed by heat requirements from April until harvest [4]. The Growth Degree Days (GDD) for each growth stage of pistachio were calculated in this study according to [7]. Briefly, the useful GDD (T > 7 °C; [40,54]) were added daily throughout the growing season starting from April.

### 4.3. Field Sampling and Aflatoxin Occurrence Data

The field sampling was performed according to European protocols for sampling (Commission Regulation (EC) No 401/2006) by the Agricultural Association of Pistachio Growers of Aegina from 2014 to 2019 between September and October. All the pistachio nuts were initially dehulled and properly dried. The determination of aflatoxin B_1_ (AFB_1_) contamination was carried out by authorized analytical laboratories that trade in food safety issues such as AgroLab RDS, SkyLab Med, and Tsakalidis Analysis and Testing. High Phase Liquid Chromatography (HPLC) with a fluorescence detector was used, and the limit of detection (LOD) was <0.1 μg/kg according to the certified external laboratories that conducted the analysis. The field data were organized in two different datasets to be used for (i) the development of the probability of AFB_1_ contamination and (ii) for the model validation, as follows: (1) 43 samples collected from 2014 to 2016, (2) 87 samples collected from 2017 to 2019.

### 4.4. Predictive Model

The relational diagram was developed and adapted based on an already existing diagram [33] and was named AFLA-pistachio (Figure 1). Briefly, the relational diagram was organized according to the principles of “systems analysis” [55]. The status of the fungus is represented by boxes, intended as state variables. The flow from one state to the following is driven by constants/parameters or intermediate variables, basically driven from weather data or crop data. The rate variables are represented by “valves”, described by mathematical functions. Variables operating in the predictive model are finally linked in a coherent mathematical framework to calculate the final output of the model—i.e., the AFB_1_ cumulative index (AFB_1_-I). AFLA-pistachio is a weather based predictive model; meteorological data (T, R and RH) were the input data used both to predict the crop phenology and *A. flavus* behavior.

### 4.5. Probability of AFB_1_ Contamination

The AFB_1_-I computed for the meteorological station and year was associated with the AFB_1_ contamination detected in the related pistachio samples, according to Battilani et al. (2013) [33] for the dataset 1 (2014–2016). Contamination data on AFB_1_ in pistachio at harvest were shared in 2 exclusive groups based on the threshold 12 μg/kg, the legal limit set for pistachio nuts to be subjected to sorting, or other physical treatment, before human consumption or use as an ingredient in foodstuffs (European Commission Regulation (EU) no. 165/2010 amending Regulation (EC) no. 1881/2006). A binary logistic regression (*p* = 1/(1 + exp⁻^(c+b*AFB1-I)^) was developed using as the dependent variable the AFB_1_ contamination of the samples and as the independent variable the AFB_1_-I generated as the output of AFLA-pistachio [33]. This approach estimates the probability of an event to occur (AFB_1_ contamination below/above the legal limit, binary data) based on an independent variable (the index given by the model at the end of the growing season). The probability (P) can range from 0 to 1; the event is considered as occurring when *p* > 0.5 and not occurring when *p* ≤ 0.5. The logistic regression module of IBM SPSS Statistics (version 25.0) was used to estimate the parameters (b and c, Table 3) of the logistic equation.

### 4.6. Validation

To confirm the ability of AFLA-pistachio to accurately predict the occurrence of AFB_1_ in pistachios, the estimated calculated probabilities were compared to the observed aflatoxin contamination field data as a measure of the goodness of fit of the predictions. The performance of the prediction was evaluated using both sets of field data; dataset 1 as the input data to estimate the parameter of logistic regression (internal validation) and the independent dataset 2 (years 2017–2019, external validation) [56].

## Figures and Tables

**Table 1:** Growth Degree Days (GDD) for the years 2014–2016, and the interval period window of the year (±days) for each growth stage of the pistachio crop.

Growth Stage	Period	2014	SE (±days)	2015	SE (±days)	2016	SE (±days)	Mean GDD	SE (±days)
Flowering	15–30 April	771	±2.1	579	±2.0	836	±2.4	729	±5.0
Pollination	1–10 May	912	±1.2	735	±1.9	987	±1.4	878	±4.7
Setting	11–20 May	1036	±1.6	877	±1.8	1114	±1.8	1009	±4.5
Early maturity	25 July–4 August	2411	±3.0	2235	±3.3	2563	±3.1	2403	±6.3
Splitting of hull	5–19 August	2690	±4.0	2527	±4.0	2852	±4.0	2690	±6.8
Harvesting	20 August–3 September	3013	±4.0	2845	±3.9	3167	±3.8	3008	±6.7

SE = Standard error.

**Table 2:** Summary data related to AFB_1_-I, the aflatoxin cumulative index generated as output by the AFLA-pistachio predictive model and aflatoxin B1 (AFB_1_ µg/kg) contamination in pistachio samples collected in Aegina Island (Greece) in the years 2014–2016 and 2017–2019, included in the 2 datasets, respectively.

Year	AFB_1_-I				AFB_1_				
	Average	Min	Max	SD	Number of Samples	Average	Min	Max	SD
Dataset 1									
2014	1014	890	1203	144.5	29	22	0.2	180	39.3
2015	1043	927	1257	122.2	11	18	0.1	73	26.1
2016	879	879	879	0.0	3	na	≤LOD	≤LOD	na
Dataset 2									
2017	900	857	1390	127.9	54	1	≤LOD	20	2.96
2018	850	837	1103	58.0	20	na	≤LOD	≤LOD	na
2019	349	349	349	0.0	13	na	≤LOD	0.5	na

na = not applicable.

**Table 3:** Parameters (b and c) and statistics of the logistic regression applied to predict the probability of having pistachio samples contaminated above 12 µg of aflatoxin B1 per kg of nuts as a function of the output of the predictive model (AFB_1_-I = aflatoxin index). The probability *p* = 0.5 corresponded to AFB_1_-I = 1153; AFB_1_-I < 1153 corresponded to *p* < 0.5 and the related pistachio samples were predicted with a contamination lower than 12 µg/kg (0 = not contaminated). On the contrary, *p* ≥ 1153 samples were considered contaminated (1= contamination above 12 µg/kg).

Parameters	Parameters Value	SE ^a^	Wald ^b^	df ^c^	Probability ^d^	Exp (b) ^e^
b	0.004	0.002	3.141	1	0.076	1.004
c	−5.131	2.595	3.911	1	0.048	0.006

^a^ SE = Standard error of each parameter; ^b^ Wald statistic is calculated for the variables in the model to determine whether a variable should be removed; ^c^ df = degrees of freedom; ^d^ probability level of parameter; ^e^ exp (b) is the factor of increase in the probability of the event when the independent variable changes by one unit.

**Table 4:** Contingency tables that summarizes the comparison between the model predictions (Predicted 0/1) and data collected in pistachio orchards (Observed 0/1). Data on aflatoxin contamination were classified in respect to the threshold of 12 µg/kg (0: <12; 1: ≥12). Probabilities ≥0.5 were considered for the contaminated samples; the contamination was underestimated when the prediction is 0 and the observed value is 1, while with prediction 1 and the observed value 0 the samples were overestimated. Dataset 1 (2014–2016) was used for internal validation and dataset 2 (2017–2019) for external validation.

Dataset 1				Dataset 2			
		Predicted				Predicted	
		0	1			0	1
Observed	0	55.8	11.6	Observed	0	95.6	3.3
	1	16.3	16.3		1	1.1	0.0

## References

[1] Elloumi O., Ghrab M., Kessentini H., Mimoun M.B. (2013). Chilling accumulation effects on performance of pistachio trees cv. Mateur in dry and warm area climate. Sci. Hortic..

[2] Afshari H., Tajabadipour A., Hokmabadi H., Moghadam M.M. (2009). Determining the chilling requirements of four Pistachio cultivars in Semnan province (Iran). Afr. J. Agr. Res..

[3] Guo L., Dai J., Ranjitkar S., Yu H., Xu J., Luedeling E. (2014). Chilling and heat requirements for flowering in temperate fruit trees. Int. J. Biometeorol..

[4] Kebour D., Mekademi K., Boutekrabt A. (2013). Test of a study of phenology of pistachio fruit (*Pistacia vera* L.) in the orchard TIGHENNIF (W. Mascara, Algeria). J. Curr. Res. Sci..

[5] Luedeling E., Brown P.H. (2011). A global analysis of the comparability of winter chill models for fruit and nut trees. Int. J. Biometeorol..

[6] Zhang J., Ranford T., Taylor C. (2015). Heat model for pistachio bloom and harvest. Sci. Hortic..

[7] McMaster G.S., Wilhelm W.W. (1997). Growing degree-days: One equation, two interpretations. Agric. Forest Meteorol..

[8] Sommer N.F., Buchanan J.R., Fortlage R.J. (1986). Relation of early splitting and tattering of pistachio nuts to aflatoxin in the orchard. Phytopathology.

[9] Doster M.A., Michailides T.J. (1994). *Aspergillus* molds and aflatoxins in pistachio nuts in California. Phytopathology.

[10] Doster M.A., Michailides T.J. (1995). The relationship between date of hull splitting and decay of pistachio nuts by *Aspergillus* species. Plant Dis..

[11] Kabirian H.R., Afshari H., Moghadam M.M., Hokmabadi H. (2011). Evaluation of pistachio contamination to *Aspergillus flavus* in Semnan Province. Int. J. Nuts Relat. Sci..

[12] Georgiadou M., Dimou A., Yanniotis S. (2012). Aflatoxin contamination in pistachio nuts: A farm to storage study. Food Control.

[13] Rushing B.R., Selim M.I. (2019). Aflatoxin B1: A review on metabolism, toxicity, occurrence in food, occupational exposure, and detoxification methods. Food Chem. Toxicol..

[14] Probst C., Njapau H., Cotty P.J. (2007). Outbreak of an acute aflatoxicosis in Kenya in 2004: Identification of the causal agent. Appl. Environ. Microbiol..

[15] Probst C., Schulthess F., Cotty P.J. (2010). Impact of *Aspergillus* section *Flavi* community structure on the development of lethal levels of aflatoxins in Kenyan maize (*Zea mays*). J. Appl. Microbiol..

[16] Cotty P.J., Jaime-Garcia R. (2007). Influences of climate on aflatoxin producing fungi and aflatoxin contamination. Int. J. Food Microbiol..

[17] Kaminiaris M., Tsitsigiannis D., Kintzios S., Mavrikou S. (2020). Pre-harvest management strategies to control aflatoxin contamination in crops. Aflatoxins: Biochemistry, Toxicology, Public Health, Policies and Modern Methods of Analysis.

[18] Doster M.A., Michailides T.J. (1999). Relationship between shell discoloration of pistachio nuts and incidence of fungal decay and insect infestation. Plant Dis..

[19] Panahi B., Khezri M. (2011). Effect of harvesting time on nut quality of pistachio (*Pistacia vera* L.) cultivars. Sci. Hortic..

[20] Aktas T., Polat R. (2007). Changes in the drying characteristics and water activity values of selected pistachio cultivars during hot air drying. J. Food Process Eng..

[21] Mahoney N.E., Gee W.S., Higbee B.S., Beck J.J. (2014). Ex situ volatile survey of ground almond and pistachio hulls for emission of spiroketals: Analysis of hull fatty acid composition, water content, and water activity. Phytochem. Lett..

[22] Beauchat L.R. (1978). Relationship of water activity to moisture content in tree nuts. J. Food Sci..

[23] Bui-Klimke T.R., Guclu H., Kensler T.W., Yuan J.-M., Wu F. (2014). Aflatoxin regulations and global pistachio trade: Insights from social network analysis. PLoS ONE.

[24] Yin Y.-N., Yan L.-Y., Jiang J.-H., Ma Z.-H. (2008). Biological control of aflatoxin contamination of crops. J. Zhejiang Univ. Sci. B.

[25] Lagogianni C.S., Tsitsigiannis D.I. (2018). Effective chemical management for prevention of aflatoxins in maize. Phytopathol. Mediterr..

[26] Tsitsigiannis D.I., Dimakopoulou M., Antoniou P.P., Tjamos E.C. (2012). Biological control strategies of mycotoxigenic fungi and associated mycotoxins in Mediterranean basin crops. Phytopathol. Mediterr..

[27] Doster M.A., Cotty P.J., Michailides T.J. (2014). Evaluation of the atoxigenic *Aspergillus flavus* strain AF36 in pistachio orchards. Plant Dis..

[28] Battilani P., Leggieri M.C. (2015). OTA-Grapes: A mechanistic model to predict Ochratoxin a risk in grapes, a step beyond the systems approach. Toxins.

[29] Maiorano A., Reyneri A., Magni A., Ramponi C. (2009). A decision tool for evaluating the agronomic risk of exposure to fumonisins of different maize crop management systems in Italy. Agric. Syst..

[30] Chauhan Y.S., Wright G.C., Rachaputi R.C.N., Holzworth D., Broome A., Krosch S., Robertson M.J. (2010). Application of a model to assess aflatoxin risk in peanuts. J. Agric. Sci..

[31] Liu C., Manstretta V., Rossi V., Fels-Klerx H.J.v.d. (2018). Comparison of three modelling approaches for predicting deoxynivalenol contamination in winter wheat. Toxins.

[32] Váňová M., Klem K., Matušinský P., Trnka M. (2009). Prediction model for deoxynivalenol in wheat grain based on weather conditions. Plant Protect. Sci..

[33] Fels-Klerx H.J.V.D., Burgers S.L.G.E., Booij C.J.H. (2010). Descriptive modelling to predict deoxynivalenol in winter wheat in the Netherlands. Food Addit. Contam..

[34] Battilani P., Camardo Leggieri M., Rossi V., Giorni P. (2013). AFLA-maize, a mechanistic model for *Aspergillus flavus* infection and aflatoxin B1 contamination in maize. Comput. Electron. Agric..

[35] Battilani P., Camardo Leggieri M. (2015). Predictive modelling of aflatoxin contamination to support maize chain management. World Mycotoxin J..

[36] Klem K., Váňová M., Hajšlová J., Lancová K., Sehnalová M. (2007). A neural network model for prediction of deoxynivalenol content in wheat grain based on weather data and preceding crop. Plant Soil Environ..

[37] Battilani P. (2016). Recent advances in modeling the risk of mycotoxin contamination in crops. Curr. Opin. Food Sci..

[38] Russelle M.P., Wilhelm W.W., Olson R.A., Power J.F. (1984). Growth analysis based on degree days. Crop Sci..

[39] Gavilán R.G. (2005). The use of climatic parameters and indices in vegetation distribution. A case study in the Spanish Sistema Central. Int. J. Biometeorol..

[40] Rahemi M., Pakkish Z. (2009). Determination of chilling and heat requirements of pistachio (*Pistacia vera* L.) cultivars. Agric. Sci. China.

[41] Salhi H., Mimoun M.B., Ghrab M. (2014). Chilling and heat requirements for flowering of the main pistachio Tunisian cultivar ‘Mateur’. Acta Hortic..

[42] Pope K.S., Brown P.H., Dose V., Silva D.D., DeJong T.M. (2014). Yield potential analysis to model dormancy requirements in pistachio. Acta Hortic..

[43] Chauhan Y., Tatnell J., Krosch S., Karanja J., Gnonlonfin B., Wanjuki I., Wainaina J., Harvey J. (2015). An improved simulation model to predict pre-harvest aflatoxin risk in maize. Field Crops Res..

[44] Rossi V., Giosuè S., Pattori E., Spanna F., Vecchio A.D. (2003). A model estimating the risk of Fusarium head blight on wheat. EPPO Bull..

[45] Abbas H.K., Zablotowicz R.M., Locke M.A. (2004). Spatial variability of *Aspergillus flavus* soil populations under different crops and corn grain colonization and aflatoxins. Can. J. Bot..

[46] Blandino M., Reyneri A., Vanara F., Pascale M., Haidukowski M., Campagna C. (2009). Management of fumonisin contamination in maize kernels through the timing of insecticide application against the European corn borer Ostrinia nubilalis Hübner. Food Addit. Contam..

[47] Champeil A., Fourbet J.-F., Doré T., Rossignol L. (2004). Influence of cropping system on Fusarium head blight and mycotoxin levels in winter wheat. Crop Prot..

[48] Blandino M., Scarpino V., Sulyok M., Krska R., Reyneri A. (2017). Effect of agronomic programmes with different susceptibility to deoxynivalenol risk on emerging contamination in winter wheat. Europ. J. Agron..

[49] Schöneberg T., Martin C., Wettstein F.E., Bucheli T.D., Mascher F., Bertossa M., Musa T., Keller B., Vogelgsang S. (2016). Fusarium and mycotoxin spectra in Swiss barley are affected by various cropping techniques. Food Addit. Contam..

[50] Leggieri M.C., Bertuzzi T., Pietri A., Battilani P. (2015). Mycotoxin occurrence in maize produced in Northern Italy over the years 2009–2011: Focus on the role of crop related factors. Phytopathol. Mediterr..

[51] González-Domínguez E., Legler S., Fedele G., Ammour M.S., Caffi T., Rossi V. Helping farmers in timing the application of biocontrol agents in viticulture. Proceedings of the Biological and Integrated Control of Plant Pathogens.

[52] Ehler L.E. (2006). Integrated pest management (IPM): Definition, historical development and implementation, and the other IPM. Pest Manag. Sci..

[53] Barzman M., Bàrberi P., Nicholas A., Birch E., Boonekamp P., Dachbrodt-Saaydeh S., Graf B., Hommel B., Jensen J.E., Kiss J. (2015). Eight principles of integrated pest management. Agron. Sustain. Dev..

[54] Kuden A.B., Kaska N., Tanriver E., Ak B.E., Tekin H. (1995). Determining the chilling requirements and growing degree hours of some pistachio nut cultivars and regions. Acta Hortic..

[55] Leffelaar P.A. (1993). Basic elements of dynamic simulation. On Systems Analysis and Simulation of Ecological Processes with Examples in CSMP and FORTRAN.

[56] Paul P.A., Munkvold G.P. (2004). A model-based approach to preplanting risk assessment for gray leaf spot of maize. Phytopathology.

